# Functional Consequences of Low Activity of Transport System A for Neutral Amino Acids in Human Bone Marrow Mesenchymal Stem Cells

**DOI:** 10.3390/ijms21051899

**Published:** 2020-03-10

**Authors:** Martina Chiu, Giuseppe Taurino, Massimiliano G. Bianchi, Erica Dander, Alessandra Fallati, Nicola Giuliani, Giovanna D’Amico, Ovidio Bussolati

**Affiliations:** 1 Department of Medicine and Surgery, University of Parma, 43125 Parma, Italy; giuseppe.taurino@studenti.unipr.it (G.T.); massimiliano.bianchi@unipr.it (M.G.B.); nicola.giuliani@unipr.it (N.G.); 2 Centro Ricerca Tettamanti, Pediatric Dept., University of Milano-Bicocca, Fondazione MBBM, 20900 Monza, Italy; e.dander@hsgerardo.org (E.D.); a.fallati@campus.unimib.it (A.F.);

**Keywords:** adaptive regulation, amino acid starvation, cell volume, glutamine, hypertonic stress, MeAIB, mesenchymal stem cells, proline, SNAT1, System A

## Abstract

In cultured human fibroblasts, SNAT transporters (System A) account for the accumulation of non-essential neutral amino acids, are adaptively up-regulated upon amino acid deprivation and play a major role in cell volume recovery upon hypertonic stress. No information is instead available on the expression and activity of SNAT transporters in human bone marrow mesenchymal stromal cells (MSC), although they are increasingly investigated for their staminal and immunomodulatory properties and used for several therapeutic applications. The uptake of glutamine and proline, two substrates of SNAT1 and SNAT2 transporters, was measured in primary human MSC and an MSC line. The amino acid analogue MeAIB, a specific substrate of these carriers, has been used to selectively inhibit SNAT-dependent transport of glutamine and, through its sodium-dependent transport, as an indicator of SNAT1/2 activity. SNAT1/2 expression and localization were assessed with RT-PCR and confocal microscopy, respectively. Cell volume was assessed from urea distribution space. In all these experiments, primary human fibroblasts were used as the positive control for SNAT expression and activity. Compared with fibroblasts, MSC have a lower SNAT1 expression and hardly detectable membrane localization of both SNAT1 and SNAT2. Moreover, they exhibit no sodium-dependent MeAIB uptake or MeAIB-inhibitable glutamine transport, and exhibit a lower ability to accumulate glutamine and proline than fibroblasts. MSC exhibited an only marginal increase in MeAIB transport upon amino acid starvation and did not recover cell volume after hypertonic stress. In conclusion, the activity of SNAT transporters is low in human MSC. MSC adaptation to amino acid shortage is expected to rely on intracellular synthesis, given the absence of an effective up-regulation of the SNAT transporters.

## 1. Introduction

Bone marrow mesenchymal stem cells (MSC) are self-renewable, multipotent cells capable of multi-lineage differentiation into adipocytes, chondrocytes or osteoblasts [1], and, hence, widely used for different applications in regenerative medicine [2,3]. Moreover, MSC secrete a variety of soluble mediators that influence various types of immune cells and have distinctive immunomodulatory properties that widen the range of their potential therapeutic applications [4,5]. Although metabolism is considered an important determinant of MSC properties, their metabolic features are yet to be completely defined [6].

In particular, metabolic features may be of particular relevance for the nutritional support that MSC can provide to other cells of the microenvironment. For example, in the case of Acute Lymphoblastic Leukemia (ALL), MSC produce and secrete asparagine (Asn) to support the growth of leukemic blasts, which are auxotroph for Asn [7,8]. Asn synthesis through asparagine synthetase requires glutamine (Gln) as the obliged nitrogen donor [9]. Thus, the nutritional support of ALL blasts by MSC is only possible if Gln availability is adequate.

Actually, the metabolic role of Gln far exceeds its participation in the synthesis of asparagine since the amino acid is needed for the synthesis of nucleotides and, through glutamate, of other non-essential amino acids. Moreover, in several types of normal and cancer cells [10], Gln also plays an anaplerotic role in sustaining cell energetics, providing carbon moieties for the TCA cycle.

Gln is the most abundant amino acid in human plasma (about 0.6 mM). However, even at this relatively high concentration, a fast influx of Gln, given its hydrophilicity, relies on the activity of transporters. Indeed, at least four families of carriers are involved in Gln uptake in mammalian cells, namely SLC1, SLC6, SLC7 and SLC38. While the exchange transporter ASCT2, coded by *SLC1A5*, has been widely found overexpressed in numerous cancer cell models, the so called System A and System N transporters, members of the SLC38 family are considered the principal unidirectional transporters for the amino acid [11]. The sodium-dependent System A transporters play important physiological functions [12]. SNAT1 and SNAT2, the most widely expressed and deeply investigated members of this group, are secondary active transporters able to build high transmembrane gradients of their substrates. System A transporters, but not other sodium-dependent amino acid carriers, are characterized by their tolerance for *N*-alkyl substitutions. For this reason, the analogue α-methylaminoisobutyric acid (MeAIB) is considered a paradigm substrate to specifically discriminate System A carrier activity [13,14,15,16,17]. Indeed, MeAIB is actively transported into SNAT1/2-positive cells and, therefore, its sodium-dependent influx can be considered an indicator of SNAT transport activity. Moreover, it is a competitive inhibitor of other substrates of SNAT1/2 and, at high concentrations, suppresses their SNAT-dependent influx, thus allowing the evaluation of SNAT1/2 contribution to the transport of substrates, such as Gln, that interact with multiple types of carriers.

No attempt to investigate Gln uptake or the expression and activity of SNAT transporters in MSC has been made yet. Here we shown that, compared with human fibroblasts, when cultured in standard growth medium, human bone marrow-derived MSC are endowed with lower activity of System A transporters, which is associated with a reduced Gln cell content and impaired ability to recover cell volume upon hypertonic stress.

## 2. Results

### 2.1. MeAIB Does Not Inhibit Glutamine Transport in Human Mesenchymal Stem Cells

**Figure 1:**
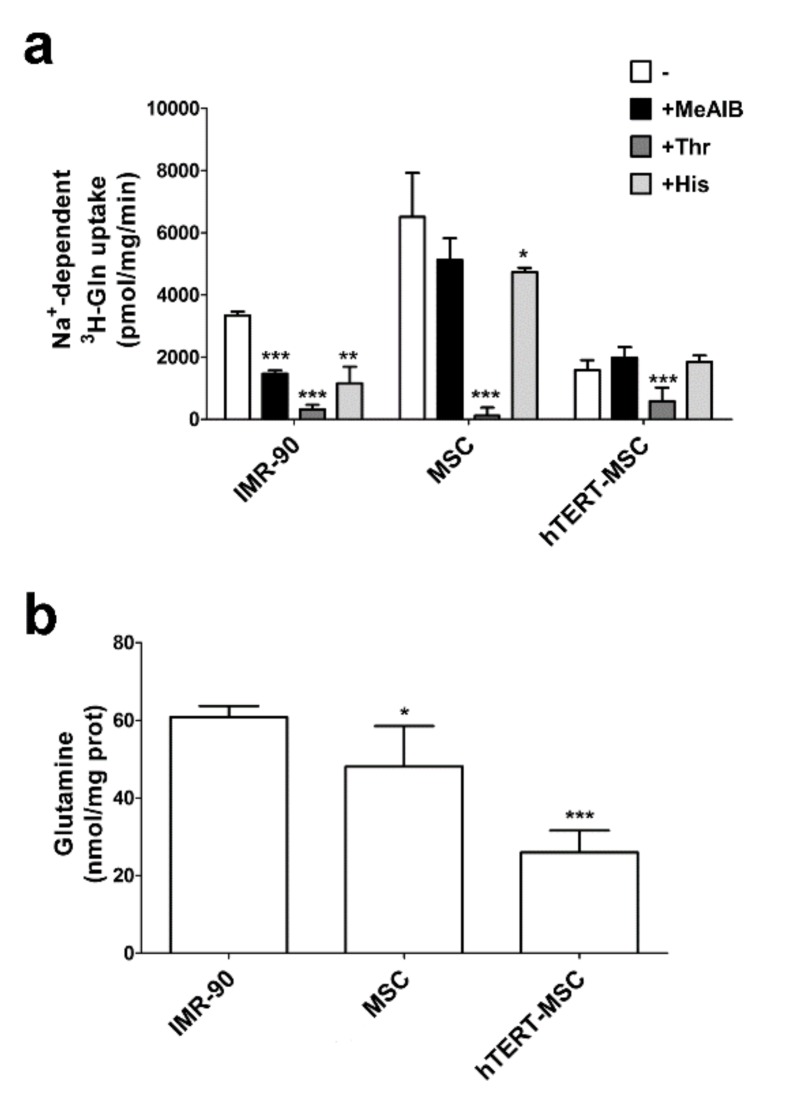
System A activity is negligible in human mesenchymal stem cells. (**a**) The 1-min uptake of ^3^H-Gln (0.1 mM, 10 μCi/mL) in IMR-90 fibroblasts, MSC and hTERT-MSC after a 90-min incubation in amino acid-free EBSS. Gln uptake was performed in EBSS in the absence (-) or in the presence of MeAIB (10 mM), threonine (Thr, 5 mM) or histidine (His, 5 mM), or in Na^+^-free EBSS. The Na^+^-dependent fraction was calculated by subtracting the uptake in the absence of Na^+^ from the Gln uptake determined in the presence of the cation under the same conditions. Data are expressed as pmol/mg prot/min and are means ± SD of two experiments, with five independent determinations each. (**b**) Intracellular Gln content in IMR-90 fibroblasts, MSC and hTERT-MSC maintained in normal growth medium. Data are expressed as nmol/mg prot and are means ± SD of two experiments with three independent determinations each. For MSC, data are the means of the data obtained from two different donors. For (**a**) and (**b**) * *p* < 0.05, ** *p* < 0.01, *** *p* < 0.001, as assessed with two-tailed Student’s *t*-tests.

Firstly, we characterized Glutamine (Gln) uptake in a strain of human primary MSC, in a human immortalized MSC line (hTERT-MSC) and in a widely employed strain of human diploid fibroblasts (IMR-90). As shown in Figure 1a, in all the cell types the Na^+^-dependent uptake of Gln was completely suppressed by a high concentration of threonine (Thr), a preferential substrate of ASCT2 [18] that also interacts at low affinity with System A [19], while a small quote of uptake was inhibited by histidine (His), a substrate of System N [20] in primary MSC and in fibroblasts. MeAIB, a specific substrate of the System A SNAT transporters [15,21], lowered Gln uptake only in fibroblasts, but neither in MSC nor in hTERT-MSC (Figure 1a). Lack of a MeAIB-inhibitable component of Gln uptake was confirmed also in the other three strains of primary MSC [22]. Despite MSC showing a higher initial Gln influx than fibroblast, the intracellular Gln content, measured in cells maintained in standard growth medium, was lower in either primary MSC or in hTERT-MSC than in IMR-90 fibroblasts (Figure 1b). This result is consistent with the absence of System A activity and suggest that in MSC Gln uptake mainly occurs through the activity of an ASCT2 exchanger transporter.

### 2.2. Mesenchymal Stem Cells Have a Low Expression of the System A Transporters

**Figure 2:**
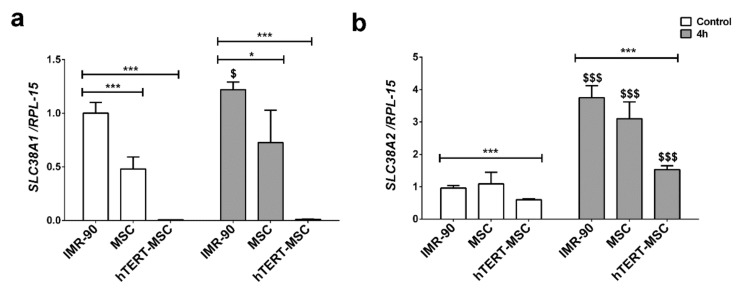
Expression of SNAT1 and SNAT2 in human fibroblasts and mesenchymal stem cells. (**a**,**b**) *SLC38A1* (**a**) and *SCL38A2* (**b**) mRNA expression was assessed by real-time PCR in IMR-90 fibroblasts, MSC and hTERT-MSC incubated in standard growth medium (empty bars) or in amino acid-free EBSS (gray bars). Data were normalized to the expression of *RPL-15*. For MSC, data were derived from cells of three different donors. Data are presented as means ± SD of two experiments with two independent determinations each. * *p* < 0.05, *** *p* < 0.001 vs. IMR-90 and $ *p* < 0.05, $$$ *p* < 0.001 vs. each control, as assessed with two-tailed Student’s *t*-tests.

The expression of SNAT1 and SNAT2, two System A transporters involved in Gln uptake, was evaluated in the three cell types. The expression of *SLC38A1*, encoding for SNAT1, was 50% lower in primary MSC than in IMR-90 fibroblasts and hardly detectable in hTERT-MSC (Figure 2a), while *SCL38A2* expression, the gene encoding for SNAT2, was comparable in primary MSC and IMR-90 fibroblasts (Figure 2b), and lower in hTERT-MSC.

It is known that the expression of SNAT transporters is regulated at different levels in several experimental conditions [12]. In particular, a distinctive feature of the SNAT2 transporter is the so-called adaptive regulation, which consists of an increase in transporter expression and activity upon amino acid starvation. Indeed, after 4 h of amino acid starvation, the expression of SNAT2 was upregulated in all the cell types tested with the highest expression in IMR-90 fibroblasts (Figure 2b), while SNAT1 expression showed a modest increase only in IMR-90 in the same conditions (Figure 2a).

**Figure 3:**
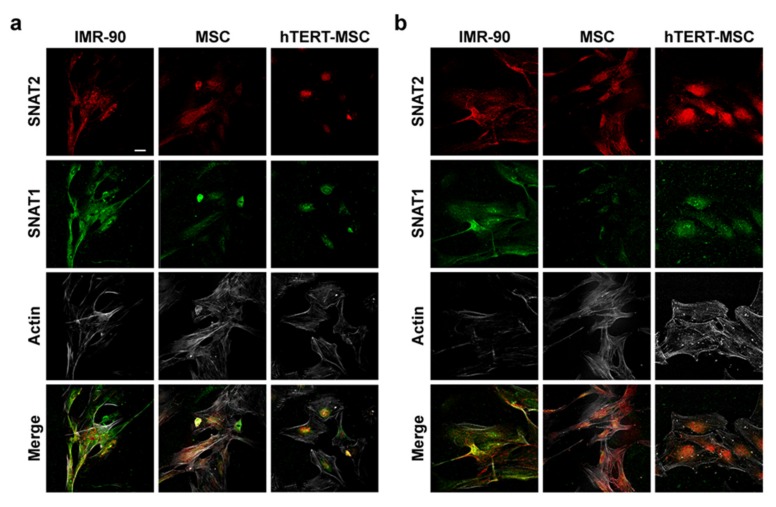
Mesenchymal stem cells have a lower membrane expression of SNAT1 and SNAT2. (**a**,**b**) Immunofluorescence of SNAT2 (red), SNAT1 (green) and actin (white) in IMR-90, MSC and hTERT-MSC incubated in complete growth medium (**a**) or in amino acid-free EBSS for 4 h (**b**). Single confocal sections of representative fields are shown. Bar = 20 μm.

Since adaptive regulation can occur at different levels, including the targeting of the transporters on the cell membrane [23], the localization of SNAT1 and SNAT2 was monitored by means of confocal microscopy, either in cells maintained in complete growth medium or starved for 4 h in the absence of amino acids, a condition associated with enhanced membrane expression and transport activity [23]. As shown in Figure 3a, in cells maintained in complete growth medium, membrane localization of both SNAT1 and SNAT2 was evident in IMR-90 cells, while only a diffuse intracellular signal is present in MSC and hTERT-MSC. Moreover, upon amino acid starvation, both SNAT1 and SNAT2 exhibited an increased membrane signal in IMR-90 cells, while the increase was barely detectable in primary MSC and not detected at all in hTERT-MSC (Figure 3b).

### 2.3. Mesenchymal Stem Cells Do Not Exhibit an Increase in System A Activity upon Amino Acid Starvation

**Figure 4:**
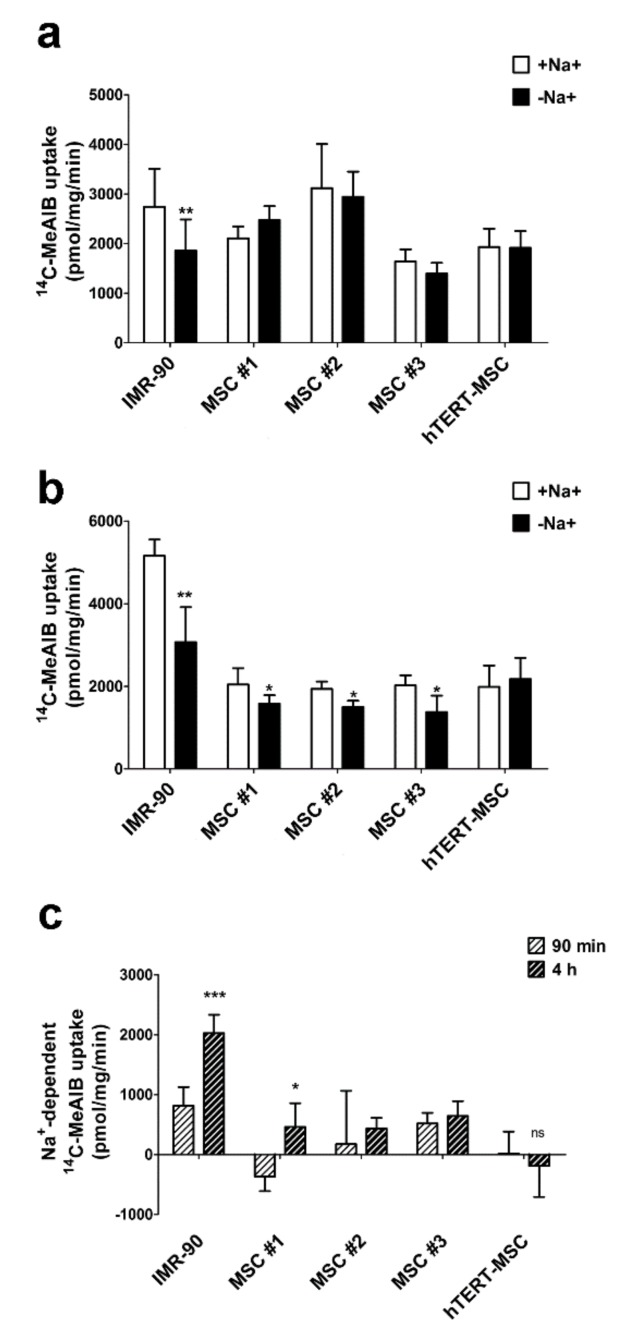
Marginal stimulation of System A activity in amino acid-starved human mesenchymal stem cells. (**a**–**c**) The 1-min uptake of ^14^C-MeAIB (0.1 mM, 2.5 μCi/mL) in IMR-90 fibroblasts, three strains of primary MSC and hTERT-MSC after a 90-min (**a**) or a 4-h (**b**) incubation in amino acid-free EBSS. MeAIB uptake was performed in EBSS in the absence (-Na^+^) or in the presence of sodium (+Na^+^). The Na^+^-dependent fraction (**c**) was calculated by subtracting the uptake in the absence of Na^+^ from the MeAIB uptake determined in the presence of the cation at the same time of incubation in EBSS. Data are expressed as pmol/mg prot/min and are means ± SD of two experiments, with five independent determinations each. * *p* < 0.05, ** *p* < 0.01, *** *p* < 0.001, as assessed with two-tailed Student’s *t*-tests.

To evaluate the functional outcomes of the different membrane expression of SNAT transporters, the uptake of their specific substrate MeAIB was evaluated in a panel of human primary MSC, in immortalized hTERT-MSC and in human fibroblasts. As expected, in non-starved cells, only IMR-90 fibroblasts displayed a Na^+^-dependent MeAIB uptake (Figure 4a). Upon 4 h of amino acid starvation, a small Na^+^-dependent component of MeAIB uptake emerged in primary MSC but not in hTERT-MSC (Figure 4b). However, while a clear-cut increment of the Na^+^-dependent component of MeAIB uptake was detectable after 4 h of starvation in fibroblasts, this effect was absent or marginal in the MSC tested (Figure 4c). Consistently, only for human fibroblasts MeAIB uptake was larger in the amino acid-starved cells than in the control cells.

### 2.4. Mesenchymal Stem Cells Do Not Show a Sodium-Dependent Proline Accumulation

**Figure 5:**
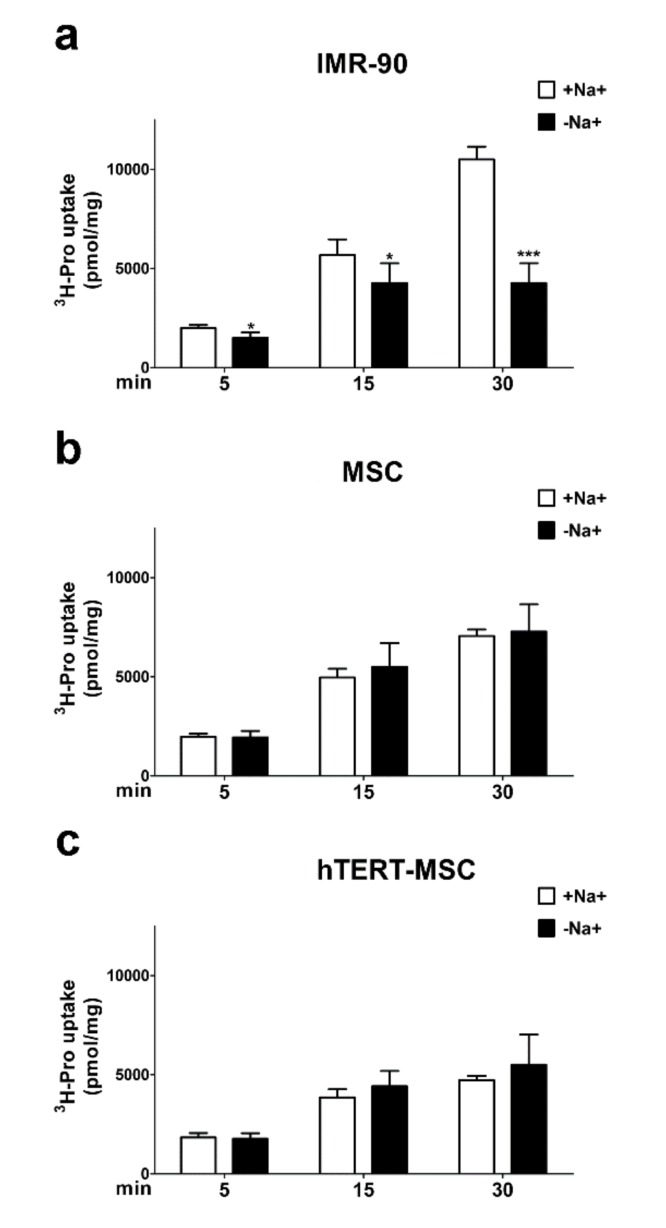
Human mesenchymal stem cells do not accumulate Pro through the activity of sodium-dependent transporters. (**a**–**c**) Uptake of ^3^H-Pro (0.1 mM, 15 μCi/mL) in IMR-90 fibroblasts (**a**), MSC (**b**) and hTERT-MSC (**c**) after a 90-min incubation in amino acid-free EBSS. Data are expressed as pmol/mg prot and are means ± SD of two experiments, with five independent determinations each. * *p* < 0.05, *** *p* < 0.001, as assessed with two-tailed Student’s *t*-tests.

Given the presence of a secondary amine in its structure, proline (Pro) cannot interact with carriers for amino acids other than System A transporters [16] and the tissue-specific SIT1 [16,24]. To further confirm the absence of System A transport activity in MSC, the time course of Pro uptake was compared in human fibroblasts and MSC. A clear-cut Na^+^-dependent uptake of Pro was already evident in fibroblasts after 5 min of incubation (Figure 5a) and steadily increased up to 30 min, while, in the absence of sodium, intracellular Pro reached a plateau at 15 min, at levels less than halved compared to cells maintained in the presence of the cation. In contrast, in both primary and immortalized MSC, no Na^+^-dependent accumulation of Pro was detected (Figure 5b,c), and the intracellular levels of the amino acid were comparable in the absence and in the presence of the cation. Moreover, MSC exhibited only a limited increase in intracellular levels of Pro from 15 to 30 min of uptake.

### 2.5. Mesenchymal Stem Cells Do Not Recover Cell Volume after Hypertonic Stress

**Figure 6:**
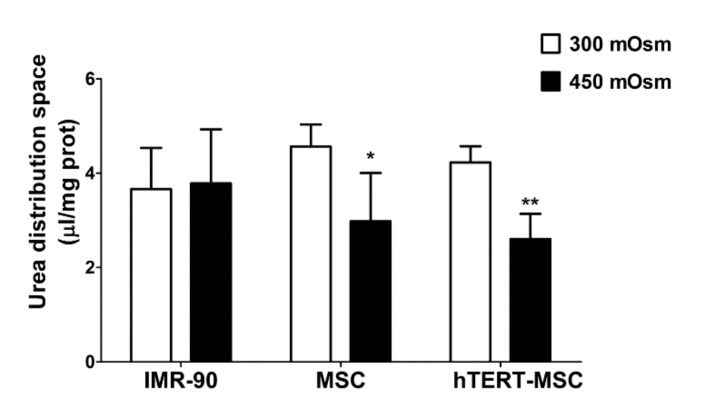
Human mesenchymal stem cells do not restore cell volume upon hypertonic stress. The 10-min distribution of ^14^C-Urea (0.5 mM, 10 μCi/mL) in IMR-90 fibroblasts, primary MSC and hTERT-MSC after a 3 h incubation with an isotonic (300 mOsm) or hypertonic medium (450 mOsm). Data are expressed as µL/mg prot and are means ± SD of two experiments, with three independent determinations each. * *p* < 0.05, ** *p* < 0.01, as assessed with two-tailed Student’s *t*-tests.

In cultured human fibroblasts [25], as well as in other human cell models [26,27,28], System A plays a pivotal role in the maintenance of cell volume under hypertonic conditions [23]. In the experiment shown in Figure 6, fibroblasts and MSC were incubated for 3 h in isotonic (300 mOsm) or hypertonic medium (450 mOsm). Then, the intracellular fluid, as a proxy of cell volume, was measured by urea distribution [29]. No difference in urea distribution was detected in IMR-90 fibroblasts, indicating an effective and complete recovery of cell volume. On the contrary, upon hypertonic stress, both primary and immortalized MSC had significantly smaller values of urea distribution space than their counterparts maintained under isotonic conditions, indicating a limited or null ability to counteract volume perturbation.

## 3. Discussion

In this report we show that bone-marrow-derived mesenchymal stem cells have a low-to-absent expression and activity of the SNAT1 and SNAT2 System A transporters, as indicated by the sodium-dependent uptake of the SNAT1/2-characterizing substrate α-methylaminoisobutyric acid (MeAIB), which is much lower than in cultured human fibroblasts. This metabolic peculiarity is associated with several functional consequences.

Glutamine is the most abundant amino acid in human plasma and play several tissue-specific functions, besides a central role in cell metabolism. The amino acid can be transported into the cells through the activity of several transporters, both sodium-dependent, such as ASCT2 and SNAT1/2, and sodium-independent, such as those of the LAT family. However, while ASCT2 and LAT are exchange transporters, SNAT1 and SNAT2 are concentrative carriers. Thus, the activity of SNAT transporters is the major determinant of intracellular glutamine levels. In turn, the intracellular glutamine concentration determines the overall size of the intracellular amino acid pool, given that it energizes the entry of essential amino acids through exchange systems (ASCT2 or LAT) [30,31], or indirectly replenishes the cysteine pool by providing glutamate that favors cystine uptake through the SLC7A11 antiporter [32]. Moreover, regulating the cell content of amino acids, SNAT transporters account for the ability of several mesenchymal cell types to recover cell volume under hypertonic conditions [25,26,27,28,33]. Upon amino acid starvation, SNAT2 [23,34] undergoes a marked up-regulation, which is an integral part of the cell response to nutritional stress [35]. As a consequence of the low expression of the SNAT transporters, these characteristic operational features of System A are hindered or even suppressed in MSC, with a blunted increase in substrate uptake upon amino acid starvation (Figure 4), a reduced ability to counteract cell shrinkage upon hypertonic stress (Figure 6) and lowered intracellular levels of glutamine at the steady state (Figure 1b).

These features are present in several strains of primary MSC from healthy donors but also in a TERT-immortalized line derived from these cells, which is endowed with the lowest expression levels of SNAT1 and SNAT2 (Figure 2a,b) among the cell types tested and, consistently, does not exhibit sodium-dependent uptake of MeAIB, even after a 4 h starvation, and any recovery of cell volume upon hypertonic incubation. Although other transporters, such as ASCT2, are expressed (results not shown) and operative (Figure 1a), they do not appear to energize amino acid accumulation with the same mechanism used by SNAT transporters and may be less efficient (see Figure 5). Thus, it is expected that, upon conditions of nutritional stress, these cells would only rely on the intracellular synthesis of substrates of system A, most of which are non-essential amino acids, without exploiting any enhancement of transport capability. This peculiarity would point to MSC as possible providers of amino acids for other cell types in a tissue undergoing amino acid restriction.

Proline (Pro) is a non-essential amino acid of particular relevance in mesenchymal cells, since it is needed for collagen synthesis and may play regulatory functions [36,37,38,39]. Due to its structural features, proline is a good substrate of SNAT transporters, which tolerate N-alkyl substitutions, but not of other sodium-dependent transporters, with the remarkable exception of the sodium- and chloride-dependent “IMINO” transporter SIT1 (SLC6A20, [24]) that, however, has a more restricted tissue-specific and development-dependent expression than SNAT transporters [40]. Limited expression and activity of SNAT transporters are associated with a reduced ability of MSC to accumulate proline from the extracellular medium (Figure 5). Due to the regulatory role of the amino acid [30,31,32,33], it would be interesting to ascertain whether changes in proline metabolism or transport occur during MSC differentiation. However, it should be noted that the amino acid is still accumulated in MSC as well as in human fibroblasts maintained in sodium-free conditions, although at reduced levels compared with fibroblasts maintained in the presence of sodium. Taking into account the values of urea distribution space provided in Figure 6, it is possible to calculate that the proline trans-membrane gradient is more than 10 in MSC and in human fibroblasts incubated without sodium, and more than 20 in fibroblasts in the presence of sodium. While the gradient measured in the presence of sodium is consistent with System A operation, the presence of a clear-cut proline gradient in the absence of sodium raises the possibility that an active, sodium-independent transporter, tentatively attributable to a member of the SLC36 family, is operative in MSC and in fibroblasts. Further experiments are needed to verify this hypothesis.

In conclusion, the results presented in this report offer a demonstration of the functional consequences of a lack of SNAT transporters in primary human cells. Moreover, they extend our knowledge on the metabolic features of MSC, possibly pointing to their role as amino acid providers under conditions of nutritional stress.

## 4. Materials and Methods

### 4.1. Cell Culture

Bone-marrow mesenchymal stem cells (MSC) were isolated as described previously [41] from healthy donors. Informed, written consent approved by the local ethical committee was obtained in all cases. hTERT-MSC were kindly provided by Dr. D. Campana (S.Jude Hospital, Memphis, TN, USA). IMR-90 lung human fetal fibroblasts were obtained from the Cell Bank of the IZSLER (Brescia, Italy). Cells, seeded at 3 × 10^3^ cells/cm^2^, were grown in low-glucose Dulbecco’s modified medium (DMEM, EuroClone) supplemented with 2 mM Gln, 10% fetal bovine serum (FBS) and antibiotics (100 U/mL penicillin and 100 μg/mL streptomycin). At Passage 3 MSC were screened for the expression of CD73, CD90, CD105, CD14 and CD34 by flow cytometry and for their ability to differentiate into osteoblasts and adipocytes. Cells were not used for more than 7 passages.

### 4.2. RT-PCR Analysis

A total of 1 μg of total RNA, isolated with the GeneJET RNA Purification Kit (Thermo Fisher Scientific, Waltham, MA, USA), was reverse transcribed with the RevertAid RT Reverse Transcription Kit (Thermo Fisher Scientific) following the manufacturer’s instruction. For real time PCR (35 cycles), cDNA was amplified with PowerUp™ SYBR™ Green Master Mix (Applied Biosystem, Foster City, CA, USA) along with the following primers (5 pmol each): *RPL-15* (for 5′-GCAGCCATCAGGTAAGCCAAG-3′, rev 5′-AGCGGACCCTCAGAAGAAAGC-3′); *SLC38A1* (for 5′-CACCACAGGGAAGTTCGTAATC-3′, rev 5′-CGTACCAGGCTGAAAATGTCTC-3′); *SLC38A2* ( for 5′-ATGAAGAAGGCCGAAATGGGA-3′, rev 5′-TGCTTGGTGGGGTAGGAGTAG-3′). Quantitative PCR was performed in a StepOneTM Real-Time PCR System (Applied Biosystems, Waltham, MA, USA). Each cycle consisted of a denaturation step at 95 °C for 30 s, followed by separate annealing (30 s, 55–58 °C) and extension (30 s, 72 °C) steps. Fluorescence was monitored at the end of each extension step. A no-template, no-reverse transcriptase control was included in each experiment. At the end of the amplification cycles a melting curve analysis was added. Data analysis was made according to the Relative Standard Curve Method [42]. Expression data were normalized to *RPL-15* mRNA expression.

### 4.3. Amino Acid Uptake

For the Gln and α-methylaminoisobutyric acid (MeAIB) influx analysis [43], 8 × 10^3^ cells/well, seeded in 96-well multi-dish plates (Falcon, Becton Dickinson Biosciences, Franklin Lakes, NJ, USA) 48 h earlier, were incubated for 90 min in Earle’s Balanced Salt Solution (EBSS, NaCl 117 mM, Tris-HCl 26 mM; KCl 5.3 mM, CaCl_2_ 1.8 mM, MgSO_4_·7H_2_O 0.81 mM, choline phosphate 0.9 mM, glucose 5.5, supplemented with 0.02% Phenol Red, adjusted at pH 7.4). This pre-incubation was aimed to minimize the trans-effects that would artificially increase the contribution of the exchange systems to transport. Cells were then rinsed with 200 μL of Na^+^-free EBSS, where NaCl was replaced by the chloride salt of *N*-methyl-d-glucamine. For Gln transport, cells were incubated for 1 min at pH 7.4 in EBSS or in a Na^+^-free EBSS, supplemented with L-(3,4-^3^H(N))-Gln (10 μCi/mL, PerkinElmer, Groningen, The Netherlands). For the discrimination of sodium-dependent transporters, the Gln influx was measured in the absence or in the presence of MeAIB (10 mM), threonine (Thr, 5 mM) or histidine (His, 5 mM), as preferential inhibitors of System A, ASCT2 or System N transporters, respectively, as described in [43].

For Pro accumulation, cells were incubated for 90 min in EBSS, rinsed with 200 μL of Na^+^-free EBSS and incubated at pH 7.4 in EBSS or in Na+-free EBSS supplemented with L-(2,3,4,5-^3^H)-Pro (15 μCi/mL, PerkinElmer).

For MeAIB initial influx, cells were incubated for 90 min or 4 h in EBSS, rinsed with 200 μL of Na^+^-free EBSS and incubated at pH 7.4 in EBSS or in a Na^+^-free EBSS supplemented with α-(1-^14^C)-MeAIB (2.5 μCi/mL, PerkinElmer).

In all cases, after the chosen uptake times, cells were washed with ice-cold urea (300 mM) to stop amino acid trans-membrane fluxes. Cells were then extracted with 50 μL of cold absolute ethanol. The extracts were mixed with 200 μL of scintillation fluid and counted with a scintillation spectrometer (Microbeta^2^, PerkinElmer, Milan, Italy). Influx data were expressed as pmol/mg prot/min while accumulation data were expressed as nmol/mg of protein.

### 4.4. Immunofluorescence

Cells were seeded on 4-chamber CultureSlides (Falcon, Becton & Dickinson Company, San Jose, CA, USA) at a density of 12.5 × 10^3^ cells/cm^2^. At the end of the treatments, cells were rinsed twice in PBS and fixed for 10 min in 3.7% paraformaldehyde in PBS. After two further rinses, cells were permeabilized with 0.1% Triton in PBS for 7 min. Cells were incubated for 1 h in blocking solution (5% of BSA, 10% of goat serum, and 0.3 M glycine in PBS), and then incubated at 4 °C overnight with anti-SNAT1 (rabbit, polyclonal, 1:300, Abcam, Cambridge, UK) or anti-SNAT2 (mouse, polyclonal, 1:300, SantaCruz Biotechnology, Dallas, TX, USA). Cells were washed with PBS and stained with Alexa Fluor 633 Phalloidin (Thermo Fisher Scientific) for 30 min. After washing, cells were incubated for 1h with Alexa Fluor 488 anti-mouse IgG and Alexa Fluor 546 anti-rabbit IgG antibodies (Invitrogen, Paisley, UK, 1:400). Cells were observed with a confocal microscope Zeiss^®^ 510 LSM Meta (Carl Zeiss SpA, Arese, Milan, Italy), using an oil 60× objective (NA 1.3).

### 4.5. Intracellular Gln Content

A total of 1 × 10^5^ cells were seeded in 12-well multi-dish plates (Falcon), and after 24 h the medium was substituted with fresh medium containing 2 mM Gln. After 18 h, cells were washed in ice-cold PBS and cell monolayers were extracted with 300 µL of absolute ethanol. Samples were then lyophilized and suspended in 250 µL of ultra-pure distilled water (Thermo Fisher Scientific). To convert glutamine into glutamate, 10 U/mL of L-asparaginase (from *E. chrysanthemi*, a gift of Jazz Pharmaceuticals) were added to the samples that were incubated for 2 h at 37 °C. Glutamate was measured with a Glutamate Assay Kit (Abcam), and the glutamine content was obtained subtracting the glutamate content of samples incubated in the absence of l-asparaginase from that of the same samples incubated with the enzyme.

### 4.6. Cell Volume

A total of 5 × 10^4^ cells were seeded in 24-well multi-dish plates (Falcon). After 24 h, the medium was substituted with complete growth medium (300 mOsm) for isotonic conditions or with DMEM supplemented with 150 mM sucrose for a final osmolality of 450 mOsm for a hypertonic condition. Osmolalities were monitored with a Wescor vapor pressure osmometer. After 3 h, the cell volume was estimated from the distribution space of the urea according to a method previously employed in cultured human fibroblasts [23]. ^14^C-Urea (10 μCi/mL, 0.5 mM final concentration) was added during the last 10 min of incubations. The experiment was stopped with two rapid washes in ice-cold 300 mM urea in water. Alcohol-soluble pools were extracted with absolute ethanol and added to scintillation fluid to be counted for radioactivity as described in Section 4.4. The results were expressed as μL/mg prot.

### 4.7. Statistical Analysis

For statistical analysis, two-tailed Student’s *t*-tests for unpaired data were used. GraphPad Prism 5.0™ was used for all the statistical analyses and *p* values <0.05 were considered statistically significant.

### 4.8. Reagents

Serum was obtained from Lonza, Basel, Switzerland. Unless otherwise stated Sigma (Milan, Italy) was the source of all the other chemicals.
